# Experimental realization of a non-magnetic one-way spin switch

**DOI:** 10.1038/s41467-019-11210-z

**Published:** 2019-07-29

**Authors:** Maren E. Mossman, Junpeng Hou, Xi-Wang Luo, Chuanwei Zhang, Peter Engels

**Affiliations:** 1 Department of Physics and Astronomy, Washington State University, Pullman, WA 99164 USA; 2 Department of Physics, The University of Texas at Dallas, Dallas, TX 75080 USA

**Keywords:** Electronic and spintronic devices, Ultracold gases

## Abstract

Controlling magnetism through non-magnetic means is highly desirable for future electronic devices, as such means typically have ultra-low power requirements and can provide coherent control. In recent years, great experimental progress has been made in the field of electrical manipulation of magnetism in numerous material systems. These studies generally do not consider the directionality of the applied non-magnetic potentials and/or magnetism switching. Here, we theoretically conceive and experimentally demonstrate a non-magnetic one-way spin switch device using a spin-orbit coupled Bose–Einstein condensate subjected to a moving spin-independent repulsive dipole potential. The physical foundation of this unidirectional device is based on the breakdown of Galilean invariance in the presence of spin-orbit coupling. Such a one-way spin switch opens an avenue for designing quantum devices with unique functionalities and may facilitate further experimental investigations of other one-way spintronic and atomtronic devices.

## Abstract (web summary)

For use in electronic and quantum applications, controlling the magnetism of a system through non-magnetic means is important. Here, the authors demonstrate a unidirectional non-magnetic spin-switch device using spin-momentum coupling in Bose–Einstein condensates of ultracold Rb-87 atoms.

## Introduction

The ability to coherently control and switch the magnetism in a system plays a central role for building next-generation electronic devices including, for example, magnetic memories and integrated circuits that rely on nonvolatile information encoded in the direction of magnetization. Current technologies generally manipulate magnetism through methods involving magnetic fields or spin-polarized currents, such as spin-transfer torque (STT)^1,2^. Although the past decade has witnessed a remarkable development in the field of STT-based spintronic devices^3^, switching the magnetism through nonmagnetic means, such as electric fields, continues to be of high interest to significantly reduce the required switching power^4^. Manipulating the magnetism, or overall spin, of a system with an electric field requires strong coupling between magnetic and electric properties and has been experimentally achieved recently in various materials including piezoelectric/multiferroic materials^5^, ferromagnetic semiconductors^6^, and van der Waals magnets^7^.

**Fig. 1:**
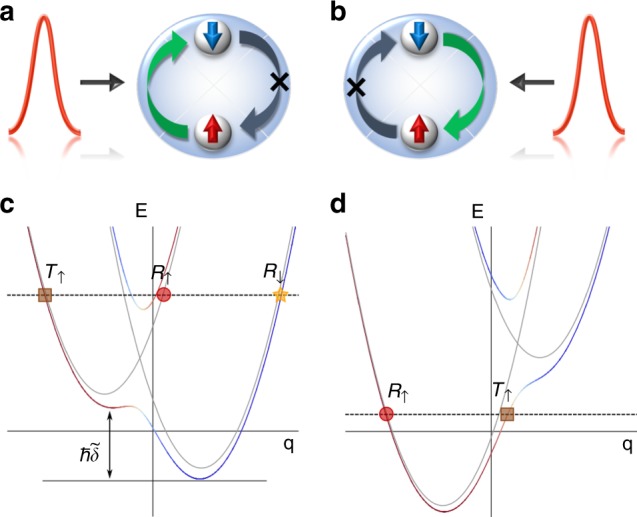
General concept and mechanism of a one-way spin switch. A control signal incoming from **a** the left or **b** the right is able to switch the spin orientation of the system from ↑ to ↓ (**a**) or ↓ to ↑ (**b**) while the reverse processes are forbidden. Sketch illustration of the spin switch and unidirectional mechanism in the comoving frame with **c** positive or **d** negative barrier velocity. Band dispersions with (red and blue) and without (gray) SO coupling are plotted. We assume an initial state with majority in spin-up state |↑〉 (red curve), labeled by the brown squares. Three channels are labeled: spin-preserved transmission (*T*_↑_), spin-preserved reflection (*R*_↑_), and spin-flipped reflection (*R*_↓_). The tilt of the dispersion represented by $\tilde \delta$ is given by a combination effect of the Raman detuning in the lab frame and the Doppler shift, proportional to the velocity of the barrier, in the comoving frame

Current nonmagnetic spin switching devices are generally not spin-orientation selective; the spin switching can occur for both spin orientations (↑ to ↓ and ↓ to ↑) and is insensitive to the orientation of external nonmagnetic potentials. Here, we introduce the concept of unidirectionality^8–10^ to spintronic devices and propose a nonmagnetic one-way spin switch, whose basic concept is illustrated in Fig. 1a, b. When a nonmagnetic control pulse interacts from the left (right), the spin orientation can be switched only from ↑ to ↓ (↓ to ↑), while the reversed process is forbidden. Such a unique unidirectionality of a spin switch may greatly enhance our ability to manipulate magnetism for designing and engineering future spintronic devices.

One-way spin switching requires a strong coupling between the direction of motion of the control potential and the spin flip in the device, which may naturally exist in a system with strong spin-orbit (SO) (that is, spin-momentum) coupling. SO coupling plays a crucial role for many condensed matter phenomena and spintronics^11^. In this context, the experimental realization of SO coupling in ultracold atomic gases^12–26^ provides a highly flexible and disorder-free platform for exploring spin-related quantum matter^27–30^ and engineering atom-based spintronic devices^31,32^. Due to the coupling between momentum and spin, Galilean invariance is broken^33,34^, indicating that the two opposite momentum directions in 1D SO coupling are no longer equivalent. The breakdown of Galilean invariance has recently been experimentally observed in a SO-coupled Bose–Einstein condensate (BEC) in an optical lattice^35^.

In this work, we utilize the breakdown of Galilean invariance and experimentally realize the conceived nonmagnetic one-way spin switch using an SO-coupled BEC (representing the device) subjected to a sweeping spin-independent repulsive Gaussian potential (representing the nonmagnetic control signal). The basic concept of such a unidirectional spin switch is illustrated in Fig. 1c, d, where the band dispersions are plotted in the comoving frame of the barrier for positive (Fig. 1c) and negative (Fig. 1d) barrier velocities ($|v_{\mathrm{b}}|\approx 2\,{\mathrm{mm}}\,{\mathrm{s}}^{-1}$). The spin switch involves two simultaneous processes: a momentum kick from the moving potential and a two-photon Raman transition induced by two counter-propagating lasers that generate SO coupling in the system. The conservation of energy and momentum in the two combined processes determines the resonant reflection and transmission channels. In the absence of SO coupling, the two spin states are decoupled, resulting only in the spin-preserved transmission channel *T*_↑_ (brown square) and reflection channel *R*_↑_ (red circle), shown in Fig. 1c. In this notation, we assume that the atoms initially are in the |↑〉 state, and the subscript indicates the majority component of the spin orientation after the barrier sweep. With SO coupling, the reflection channel on the lower SO-coupled band *R*_↓_ (orange star) reverses the spin for positive barrier velocities as illustrated in Fig. 1c. This same spin switching channel does not exist for negative barrier velocities as shown in Fig. 1d, yielding unidirectionality in the system. We explore such a unidirectional spin-switch mechanism in detail experimentally and theoretically below and observe that the efficiency of the spin switch strongly depends on the barrier sweeping velocity and mean field interactions present between the atoms in the BEC. We find that the experimental results agree well with numerical simulations based on mean field theory and confirm the intuitive explanation of the spin-switch mechanism given above.

## Results

### Experimental realization of a unidirectional spin switch

**Fig. 2:**
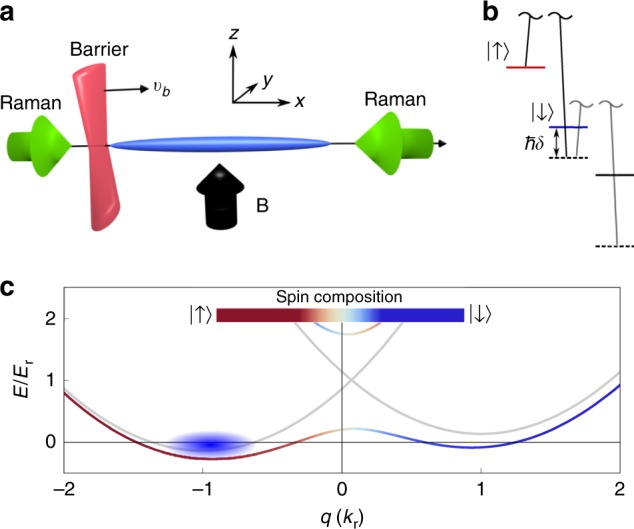
Experimental setup. **a** Two counter-propagating Raman beams applied along *x* induce SO coupling along the elongated axis of the BEC. A repulsive, spin-independent optical barrier is initially positioned outside of the BEC and then swept along the *x*-axis at a constant velocity, $v_{\mathrm{b}}$. **b** Two pseudospin states, *m*_F_ = −1 (≡|↑〉) and 0 (≡|↓〉), are Raman coupled with coupling strength Ω and detuning *δ*, while the *m*_F_ = +1 state is effectively decoupled from the system. **c** The resulting SO-coupled double-well band dispersion for parameters ℏΩ = 1.53*E*_r_, ℏ*δ* = 0.27*E*_r_. Colors of band represent the spin composition of the dressed states, where red (blue) represents the |↑〉 (|↓〉) spin state. The blue shaded oval in the left dispersion minimum represents the initial state and quasimomentum of the BEC

In the experiment, a BEC composed of 7 × 10^5 87^Rb atoms is confined in an elongated optical dipole trap with an aspect ratio of ~100:1 (Fig. 2a). For reference, the longitudinal speed of sound in the center of the BEC is *c*_*s*_ ≈ 2.2 mm s^−1^. A 10 G uniform magnetic bias field is applied in the *z* direction, splitting the internal atomic hyperfine ground state |*F* = 1〉 into three discrete states (Fig. 2b). Two counter-propagating Raman beams coherently couple the |↑〉 ≡ |*F* = 1, *m*_F_ = −1〉 and |↓〉 ≡ |*F* = 1, *m*_F_ = 0〉 states, inducing SO coupling in the system with Raman coupling strength ℏΩ = 1.53 *E*_r_ and Raman detuning ℏ*δ* = 0.27 *E*_r_, where $E_{\mathrm{r}} = (\hbar ^2k_{\mathrm{r}}^2)/2m$ is the recoil energy and we defined the recoil momentum *k*_r_ = 2*π*/*λ*_r_. For more information on the generation of the SO-coupled system, see “Methods”. The resulting dispersion features a double-well structure, shown in Fig. 2c for the provided experimental parameters. With a suitable Raman laser detuning, the atoms are prepared in the left (red) well, with the state of the atoms having a majority amplitude in the |↑〉 state (Fig. 2c). A spin-independent barrier with a potential height of *U*_b_ = 8.3 *E*_r_, ~15 times the chemical potential of the system, acts as the control signal for the spin switch and is swept through the BEC at a constant velocity, $v_{\mathrm{b}}$.

**Fig. 3:**
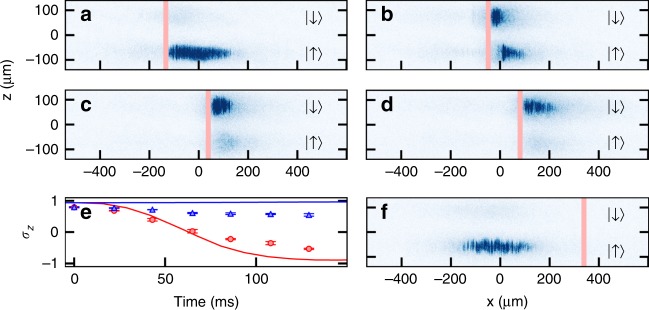
Progression of dynamics during a barrier sweep. **a**–**d** The potential barrier, represented by the red vertical line, moves to the right at $v_{\mathrm{b}}=2\,{\mathrm{mm}}\,{\mathrm{s}^{-1}}$. Atoms initially occupying the |↑〉 state are transferred to the |↓〉 state as the sweep progresses. Experimental absorption images are taken after 10.1 ms time-of-flight, during which a Stern–Gerlach imaging technique is used to vertically separate the individual spin states. Images correspond to times **a** *t* = 22 ms, **b** *t* = 87 ms, **c** *t* = 130 ms, and **d** *t* = 195 ms into the sweep. **e** Spin polarization during the sweep. Data points are mean ± s.d. for five experimental runs at each measured time for a barrier moving at $v_{\mathrm{b}}=+2\,{\mathrm{mm}}\,{\mathrm{s}^{-1}}$ (red circles) and $v_{\mathrm{b}}=-2\,{\mathrm{mm}}\,{\mathrm{s}^{-1}}$ (blue triangles). Red and blue solid curves are numerical Gross–Piteavskii equation (GPE) simulations for right and left moving barriers, respectively. **f** BEC remains in the |↑〉 state after a +15 mm s^−1^ full barrier sweep

Experimental images of the atomic density, like those shown in Fig. 3, are taken as the barrier is swept through the SO-coupled BEC. Absorption images are taken after 10.1 ms time-of-flight during which a Stern–Gerlach kick vertically separates the different spin components. The evolution of the spin polarization, defined as *σ*_*z*_ = (*N*_↑_ − *N*_↓_)/*N*_total_, is tracked with respect to time during a $v_{\mathrm{b}}=\pm 2\,{\mathrm{mm}}\,{\mathrm{s}^{-1}}$ sweep in Fig. 3e (see also Supplementary Note 1). Here, *N*_↑_ and *N*_↓_ are the atomic populations in the spin |↑〉 and |↓〉 states, respectively, and *N*_total_ = *N*_↑_ + *N*_↓_. At this featured velocity for a barrier traveling in a positive direction of motion (+*x*), the BEC is reflected from the potential barrier and its spin polarization is gradually flipped from |↑〉 to |↓〉 (Fig. 3a–d and Supplementary Fig. 1). For large positive velocities, such as $v_{\mathrm{b}}=15\,{\mathrm{mm}}\,{\mathrm{s}^{-1}}$ in Fig. 3f, the BEC is fully transmitted through the potential barrier without undergoing a spin flip. For a barrier moving in the opposite (−*x*) direction, the overall spin of the system is nearly constant across all tested velocities, indicating that the barrier cannot switch the spin while traveling in this direction. The slight decrease of the experimentally observed spin polarization in Fig. 3e is due to heating from the Raman beams, and is discussed in Supplementary Note 1.

**Fig. 4:**
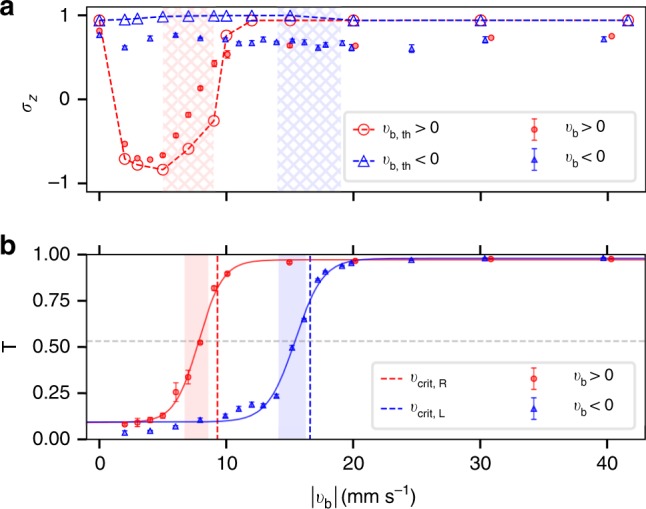
Properties of unidirectional spin switch. **a** Spin polarization versus $v_{\mathrm{b}}$ for positive (red filled circles) and negative (blue filled triangles) barrier velocities. Plotted data are mean ± s.d. for five experiments at each velocity. Hatched areas indicate velocity ranges where heating was experimentally observed. Numerical results for positive (red empty circles) and negative (blue empty triangles) barrier velocities are connected by dashed lines. **b** Transmission coefficient versus $v_{\mathrm{b}}$. Solid lines are sigmoidal fits to data and the shaded regions indicate the area where the sigmoidal fit rises from 30% to 70%. The horizontal gray-dashed line indicates the inflection point of both fits at *T* = 0.53. GPE numerical simulations without interactions result in sharp transitions between total reflection and total transmission (instead of a crossover region). The crossover velocity for each transition in the noninteracting case is represented by a vertical-dashed line

The final spin polarization, *σ*_z_, and the transmission coefficient, *T* = *N*_T_/*N*_total_, are measured after the barrier has been swept through the BEC for a large range of $v_{\mathrm{b}}$, where *N*_T_ is the atomic population behind the barrier at the end of a sweep. Experimental and numerical results are presented in Fig. 4. Three distinct regions for a barrier moving in the +*x* direction (red data points) emerge:

(i) Low barrier velocity ($0\,{\mathrm{mm}}\,{\mathrm{s}}^{ - 1} < \, v_{\mathrm{b}} \, \lesssim \, 4$ mm s^−1^). In this region, the atoms are strongly reflected from the moving barrier (*T* ≈ 0). When the barrier velocity exceeds a lower critical velocity of $v_{\mathrm{b}}=1.1\,{\mathrm{mm}}\,{\mathrm{s}^{-1}}$ (see “Discussion” below and Supplementary Notes 2 and 3), the spin polarization decreases, reaching a minimum at $v_{\mathrm{b}}\approx 4\,{\mathrm{mm}}\,{\mathrm{mm}}\,{\mathrm{s}^{-1}}$, where the spin-switch efficiency, *η*_s_ = (*σ*_*z*,max_ − *σ*_*z*,min_)/2, is experimentally found to be 85.8 ± 0.8% (*σ*_*z*,min_ = −0.72 ± 0.02).
(ii) Medium barrier velocity ($4\,{\mathrm{mm}}\,{\mathrm{s}}^{ - 1} \, \lesssim \, v_{\mathrm{b}} \, \lesssim \, 11$ mm s^−1^). In this region, the BEC is partially transmitted and partially reflected by the moving barrier. As the barrier velocity increases, the spin polarization increases, reaching a plateau near $v_{\mathrm{b}} \approx 11 \, {\mathrm{mm}} \, {\mathrm{s}}^{-1}$. This region, where the BEC is transitioning between reflection and transmission, is accompanied by heating in the experiment, depicted by hatched regions in Fig. 4a. The transmission coefficient for a right-moving barrier can be fit by a sigmoidal function centered around $7.8_{ - 0.8}^{ + 1.1}$ mm s^−1^ (solid red line in Fig. 4b). In the noninteracting case, numerical simulations instead indicate that a sharp transition occurs at a crossover velocity $v_{{\mathrm{co}}}^{\mathrm{R}} \cong 9.3$ mm s^−1^, represented by a red vertical-dashed line in Fig. 4b (see “Methods” and Supplementary Note 2).
(iii) High barrier velocity ($v_{\mathrm{b}} \gtrsim 11$ mm s^−1^). In this region, the BEC is transmitted through the barrier without reflection (*T* ≈ 1). The spin polarization plateaus at a constant value comparable to the initial spin polarization without a sweeping barrier (*t* = 0), indicating that no spin switch occurs at high velocities.

For a left moving barrier, three similar regions can be distinguished using transmission coefficient results, as no significant change of spin polarization is detected in this direction. For a barrier traveling in −*x* direction, region (i) ends at $v_{\mathrm{b}}\approx -14\mathrm{mm}\,{s^{-1}}$ and region (iii) begins at $v_{\mathrm{b}} \lesssim - 21$ mm s^−1^. The transmission coefficient in region (ii) can be fit to a sigmoidal function centered at $15.4_{ - 1.3}^{ + 0.8}$ mm s^−1^ (solid blue line in Fig. 4b), where heating is again observed within this region during the experiment (blue hatched region in Fig. 4a). In the noninteracting case, numerics reveal that another sharp transition occurs at a new crossover velocity $v_{{\mathrm{co}}}^{\mathrm{L}} \cong 16.6$ mm s^−1^, represented by a blue vertical-dashed line in Fig. 4b. These crossover velocities can be analytically calculated in the noninteracting regimes (see “Methods” and Supplementary Note 2) and are found to be robust with respect to parameters such as trapping frequencies and atom numbers. Experimentally observed heating around the crossover regimes is not captured by the Gross–Piteavskii equation (GPE) simulations and is closely related to dynamic instabilities and finite-temperature effects not incorporated in the GPE. This will be discussed further below in the context of the role of interactions in the system. The above results show that our experimental setup successfully implements a nonmagnetic one-way spin switch.

### Spin-switch mechanism

**Fig. 5:**
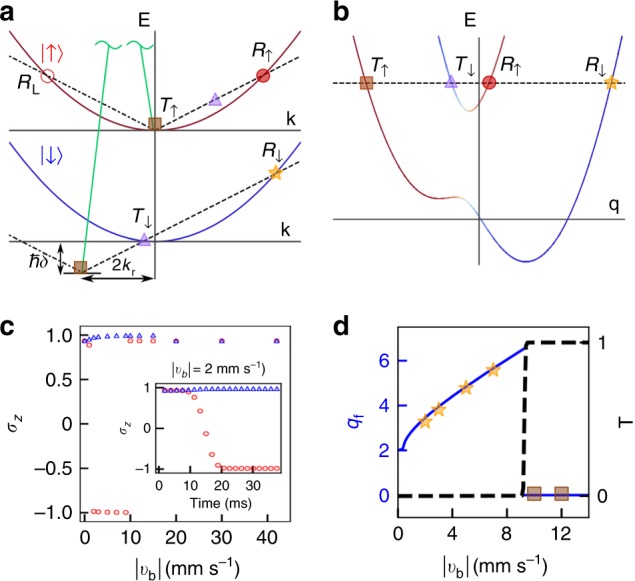
Unidirectional spin-switch mechanism. Illustration of the coupling channel mechanism in the **a** uncoupled laboratory frame and in the **b** SO-coupled comoving frame with positive (black-dashed line) or negative (black dot-dashed line) barrier velocity. Atoms prepared in the |↑〉 spin state are coupled to the |↓〉 spin state through a Raman transition (green solid lines). Three channels emerge in the uncoupled system: *T*_↑_ (brown squares), *R*_↑_ (red filled circle), *R*_L_ (red open circle); and three channels in the spin-orbit-coupled system: *T*_↑_ (brown squares), *T*_↓_ (purple triangles), *R*_↓_ (orange star). **c** Noninteracting GPE simulation of spin polarization versus $v_{\mathrm{b}}$ (interacting case in Fig. 4a). The inset shows the evolution of spin polarization during a highly efficient $v_{\mathrm{b}}=2\,{\mathrm{mm}}\,{\mathrm{s}^{-1}}$ sweep, corresponding to the interacting case in Fig. 3e. **d** Final quasimomentum *q*_f_ (solid blue curve), and transmission coefficient T (black dashed), calculated from theory for a barrier with $v_{\mathrm{b}} > \,{0}$. Results from GPE simulations (orange stars and brown squares) indicate the resonant channels at these velocities

The spin switch relies on momentum and energy transfer during two simultaneous processes: a kick imparted by a moving barrier and a Raman transition. In the laboratory frame, a moving Gaussian barrier depends on both space and time, yielding the Fourier spectrum,

1 $$\tilde V_{\mathrm{b}}({\mathrm{\Delta }}k,\omega ) = {\cal{F}}({\mathrm{\Delta }}k)\delta ({\mathrm{\Delta }}kv_{\mathrm{b}} - \omega ),$$

where *δ*(*x*) is the Dirac delta function. The coefficient function

$${\cal{F}}({\mathrm{\Delta }}k) = U_{\mathrm{b}}\sqrt {\pi /2w_{\mathrm{b}}^2} \,{\mathrm{exp}}\,( - ({\mathrm{\Delta }}k)^2w_{\mathrm{b}}^2{\mathrm{/}}8 - i{\mathrm{\Delta }}kx_0)$$

depends on the barrier profile, where *U*_b_, *w*_b_, and *x*_0_ are the barrier height, width, and initial position, respectively. The Dirac delta function requires that the energy and momentum transfer satisfies the given relation *ω* = Δ*kv*_b_, represented by diagonal-dashed and dot-dashed lines in Fig. 5a with slopes of $\pm v_{\mathrm{b}}$. Figure 5a illustrates the spin-switch mechanism in the uncoupled laboratory frame. Energy and momentum conservation in the system leads to the resonance conditions *E*_i_ = *E*_f_ − ℏ*ω* and *q*_i_ = *q*_f_ − Δ*k*, where the subscripts i (f) indicate the initial (final) state of the energy and quasimomentum during a barrier sweep. In the SO-coupled picture, quasimomentum is related to the noncoupled kinetic momentum through the relation *q*_i(f),*σ*_ = *k*_i(f),*σ*_ ± *k*_r_, where (−) is used for the *σ* = |↑〉 and (+) is used for *σ* = |↓〉 spin states.

In the absence of SO coupling, atoms are initialized to have zero kinetic momentum and energy. If prepared in the |↑〉 state at the band minimum (brown square in the upper branch of Fig. 5a), the atoms will remain in the spin-up subspace. From the above Dirac delta requirement, the resonance condition can be written as $\frac{{\hbar k_{\mathrm{f}}^2}}{{2m}} = {\mathrm{\Delta }}kv_{\mathrm{b}}$, where *k*_f_ = Δ*k*. This condition results in the solutions $\hbar k_{f}=2mv_{\mathrm{b}}$ or ℏ*k*_f_ = 0, which are given by the points of intersection between the spin-up dispersion band and the dashed (dot-dashed) line for a right (left) sweeping barrier, indicated by *R*_↑_ (red filled circle) and *R*_L_ (red open circle) in Fig. 5a, respectively. These reflection channels do not result in a spin flip.

In the presence of SO coupling, the atoms experience an additional momentum kick via a two-photon Raman transition. Atoms prepared in the |↑〉 spin state with *k*_i_ = 0 and *E*_i_ = 0 can undergo a two-photon transition into the |↓〉 state under the right conditions. The corresponding resonance condition becomes $\frac{{\hbar k_{\mathrm{f}}^2}}{{2m}} + \delta = {\mathrm{\Delta }}kv_{\mathrm{b}}$, where *k*_f_ = Δ*k* − 2*k*_r_ now includes the two-photon Raman momentum kick and *δ* is the SO coupling detuning. Two spin-flip resonance channels, given by the intersection points between spin-down band dispersion and the dashed line, emerge in this case for the right-moving barrier, shown in Fig. 5a. The transmission channel *T*_↓_ (purple triangles) exists at small negative final momentum, and the reflection channel *R*_↓_ (orange star) exists at large positive final momentum.

The unidirectionality of the spin switch described in this work manifests itself through nonexistent resonance channels when the barrier is swept in the opposite direction. In this case, atoms initialized in the |↑〉 state are driven along the black dot-dashed line in Fig. 5a and will remain in the spin-up subspace, with and without SO coupling. Analogous arguments apply when the initial spin orientation and the direction of the barrier motion are reversed.

To understand the channel preference, we move into a comoving frame with respect to the barrier and investigate the SO-coupled band dispersion. The initial quasimomentum and detuning in the comoving frame are adjusted such that $q_{{\mathrm{i,cm}}} = q_{\mathrm{i}} \pm \frac{{|v_{{\mathrm{b}}|}}}{{v_{\mathrm{r}}}}k_{\mathrm{r}}$ and $\hbar \delta _{{\mathrm{cm}}} = \hbar \delta \pm \frac{{4|v_{{\mathrm{b}}|}}}{{v_{\mathrm{r}}}}E_{\mathrm{r}}$, respectively, for either a left (+) or a right (−) moving barrier. The change in detuning is due to the Doppler shift, scaled with recoil velocity $v_{\mathrm{r}}=\hbar k_{r}/m$. This is schematically presented in Fig. 5b for $v_{\mathrm{b}}\approx2\,{\mathrm{mm}}\,{\mathrm{s}^{-1}}$. Resonance channels indicated by the same color and shape as in Fig. 5a can exist where the dashed line and band dispersion intersect. In the presence of SO coupling and for $v_{\mathrm{b}} \, \gtrsim \, 1.1\,{\mathrm{mm}}\,{\mathrm{s}}^{ - 1}$, the resonance channels *R*_↑_ and *T*_↓_ are closed due to an avoided band crossing (see Fig. 5d). Instead, atoms prefer the transmission channel *T*_↑_ or reflection channel *R*_↓_ above or below a critical barrier velocity, respectively. This is confirmed by numerical simulations shown in Fig. 5c, d. For barrier velocities below $v_{\mathrm{b}} \approx 1.1\,{\mathrm{mm}}\,{\mathrm{s}^{-1}}$, the barrier cannot drive the atoms over the band barrier around *q* = 0 in the SO-coupled dispersion, resulting in no spin flip at sufficiently low barrier velocities (see Supplementary Note 1 and Supplementary Fig. 5 for more details).

### The role of interactions

To elucidate the effects of interactions, numerical simulations of the GPE are conducted for a range of sweeping velocities in the presence of interactions, as included in Figs. 3e, 4 and Supplementary Figs. 1b and 2a. These results are found to be consistent with experimental observations. For speeds at which the BEC is fully transmitted through the barrier, the momentum maintains a narrow distribution from the initial state. As the speed of the barrier decreases and an increasingly larger fraction of the condensate is reflected by the moving barrier, a spin flip process occurs for positive velocities, despite the spin independence of the potential. Momentum-space analysis reveals that the momentum distribution of the resulting BEC is shifted and split for slow barrier velocities, leading to rapid density modulations in real space due to the superposition of different plane wave states. For detailed momentum-space analysis, see Supplementary Note 1.

We note that in the single particle regime, the density profiles and momentum spaces profiles are smooth, ideal Gaussian-like curves. A sharp spin-switch transition and ~100% spin-switch efficiency are numerically observed in the noninteracting regime. (see Fig. 5c and Supplementary Note 2). In the interacting case, the spin polarization has a smooth crossover region, which involves the superposition of multiple momentum states for the final state. Interactions broaden the transition from reflection to transmission at respective crossover velocities, but the GPE simulations are not able to capture the experimentally observed heating in the system. While Raman-induced heating persists for all sweeping speeds, the enhanced heating in the crossover region can be ascribed to two effects: finite temperature and dynamic instabilities^36^. As evident in our GPE simulations, multiple excitations are present in momentum space near the crossover velocities, which are the signature of dynamic instabilities induced by complex Bogoliubov excitation spectra, leading to significant atom loss over time. Finite-temperature effects, which are presumed to originate from the coupling between the condensate wavefunction and the thermal atoms, are also enhanced in the crossover region due to an increased number of scattering channels available in phase space. Although the zero-temperature GPE does not account for thermal atoms or other heating effects, it is able to successfully capture the key features of the unidirectional spin switch.

## Discussion

Our numerics show that in the absence of interactions, a spin-switch efficiency of nearly 100% can be achieved. In our experimental implementation, the efficiency of the spin switch could therefore be improved by reducing the atomic interaction, for example through smaller atomic number, weaker confinement along the axial direction, Feshbach resonances, etc. In addition to this, since the spin-switch mechanism relies on single particle theory, it may also apply to SO-coupled degenerate Fermi gases, given that the initial momentum and energy distributions of the atoms satisfy the energy and momentum resonance conditions. Similar unidirectional spin switches may also be engineered in electronic materials, for instance in spin-orbit coupled nanowires with electrically controlled moving potential pulses. For future studies, it would be intriguing to investigate the one-way spin switch in a 2D SO-coupled system (for example, in a system subjected to Rashba coupling) that has been realized in experiments for both Bose and Fermi gases^19,20^. In summary, our proposed concept of a unidirectional spin-switch device and its experimental realization opens an avenue for designing innovative quantum devices with unique functionalities and may facilitate further experimental investigations of other one-way spintronic and atomtronic devices.

## Methods

### Experimental preparation

Our experiments begin with a BEC consisting of 7 × 10^5 87^Rb atoms confined in an elongated optical dipole trap with trap frequencies {*ω*_*x*_, *ω*_*y*_, *ω*_*z*_} = 2*π* × {3.07, 278, 278} Hz, where the atoms have been prepared in the |*F*, *m*_F_〉 = |1, −1〉 state. Two counter-propagating Raman beams (*λ*_r_ = 789.1 nm), applied along the *x* direction, coherently couple the |1, −1〉 and the |1, 0〉 states, while the |1, +1〉 state is decoupled due to the quadratic Zeeman shift. This generates an effective pseudospin-1/2 system with |↑〉 ≡ |1, −1〉 and |↓〉 ≡ |1, 0〉.

To dress the atoms with SO coupling, the Raman beams are first linearly ramped from zero to a final coupling strength of ℏΩ = 1.53 *E*_r_ at a large Raman detuning of *δ*_0_ ≈ 20 kHz. The detuning of the Raman drive is then subsequently reduced to a final value of *δ* = 0.27 *E*_r_/*h* = 1 ± 0.030 kHz in 100 ms.

While preparing the atoms, a repulsive barrier is jumped on either to the left or right of the BEC (see Fig. 2a). This barrier is generated by a laser propagating in the *z* direction, with wavelength *λ*_b_ = 660 nm, and Gaussian widths of {*w*_*x*_, *w*_*y*_} = {11, 63} μm. This laser creates a repulsive potential of height *U*_b_ = 8.3 *E*_r_ ≈ 15*μ*, where *μ* is the chemical potential at the center of the BEC. Once the atoms in the |↑〉 state are dressed with SO coupling, a mirror galvanometer is used to sweep the repulsive barrier through the BEC along the *x* direction at different constant velocities *v*_b_.

After the sweep, all optical fields are jumped off and absorption imaging is performed along the *y* direction after 10.1 ms time-of-flight expansion, during which a Stern–Gerlach technique is used to spatially separate the two spin components. Atoms occupying the |↓〉 or |↑〉 state are clearly separated during the imaging process. For this experimental setup, sweeping in the +*x* (−*x*) direction is associated with the +*q *(−*q*) direction in the SO-coupled dispersion.

In addition to data where the barrier is swept through the BEC, data were recorded where no sweep occurred to understand heating effects due to the presence of the Raman beams. Figure 4a of the main text shows an initial spin polarization (at *t* = 0) of *σ*_*z*,0ms_ = 0.818 ± 0.015. As the atoms are held in trap in the presence of SO coupling, the Raman beams induce heating. The longest time over which the atoms are held in the dressed state is 258 ms as needed to complete a full sweep with $v_{\mathrm{b}}=2\,{\mathrm{mm}}\,{\mathrm{s}^{-1}}$. Holding the atoms for this amount of time results in a reduced spin polarization of *σ*_*z*,258ms_ = 0.450 ± 0.044. Some of the discrepancies between theoretical predictions and experimental results is thus attributed to background contamination of the images from residual thermal atoms created by heating from the Raman beams. For example, a 10% thermal background population with respect to the total number of atoms in the system results in the spin polarization being reduced from *σ*_*z*_ to 0.82*σ*_*z*_.

### GPE numerics

The experimental system and its dynamics are described by the GPE,

2 $$i\frac{\partial }{{\partial t}}{\mathrm{\Psi }} = \left( {H_0(x) + V_{\mathrm{b}}(x,t) + \frac{g}{2}|{\mathrm{\Psi }}|^2} \right){\mathrm{\Psi }},$$

under the mean-field approximation, where Ψ = (*ψ*_↓_, *ψ*_↑_)^*T*^ is the two-component wave function. The interaction strength between the two spin components in the system is given by *g*_↓↓_ = *g*_↓↑_ = *g*_↑↓_ = 0.9954*g*_↑↑_ for ^87^Rb, with the corresponding effective 1D interaction strength *g*_↑↑_ = 1426 *E*_r_, due to wavefunction normalization in the simulations. The time-independent, single particle Hamiltonian for particles dressed with SO coupling is given by

3 $$H_0(x) = \left( {\begin{array}{*{20}{c}} {\frac{{(\hbar k)^2}}{{2m}} - \frac{{\hbar \delta }}{2}} & {\frac{{\hbar \Omega }}{2}e^{ - i2k_{\mathrm{r}}x}} \\ {\frac{{\hbar {\mathrm{\Omega }}}}{2}e^{i2k_rx}} & {\frac{{(\hbar k)^2}}{{2m}} + \frac{{\hbar \delta }}{2}} \end{array}} \right) + \frac{1}{2}m\omega _x^2x^2,$$

where *ω*_*x*_ is the trapping frequency along *x*. Finally, the space and time-dependent potential is described by an optical Gaussian beam,

4 $$V_{\mathrm{b}}(x,t) = U_{\mathrm{b}}\,{\mathrm{exp}}\,( - 2(x - (x_0 - v_bt))^2{\mathrm{/}}w_b^2),$$

where *U*_b_ is the height, *x*_0_ is the initial position, $v_{\mathrm{b}}$ is the sweeping speed, and *w*_b_ = *w*_*x*_ denotes the barrier width.

### Crossover velocity

The crossover velocities can be determined using a noninteracting, single particle model according to the following method. With no known analytic solution for the scattering of a single particle by a Gaussian barrier, we consider a more treatable potential with a similar real-space profile (see inset of Supplementary Fig. 4):

5 $$V_{\mathrm{b}} \prime (x) = U_{{\mathrm{b}}\prime } / {\mathrm{cos}}h^2(x {/} w_{{\mathrm{b}}\prime }),$$

where $U_{\mathrm{b}\prime}$ and ${\mathrm{w}}_{\mathrm{b}\prime}$ are the potential height and width of the modified barrier profile. This potential reproduces the GPE results shown in Fig. 5a. Details concerning the derivation of the crossover velocities can be found in Supplementary Note 2.

## Supplementary information

Supplementary Information

## Data Availability

All relevant code used for numerical studies in this work is available from the corresponding authors on reasonable request.

## Data Availability

All relevant experimental and numerical datasets in this work will be made available from the corresponding authors upon reasonable request.
