# Supplementary material for: Experimental realization of a non-magnetic one-way spin switch
Source: Nat Commun. 2019 Jul 29;10:3381. doi: 10.1038/s41467-019-11210-z (PMC6662681; doi:10.1038/s41467-019-11210-z)
Supplement: Supplementary file 1 — Supplementary Information [file 41467_2019_11210_MOESM1_ESM.pdf]

Experimental realization of a non-magnetic one-way spin switch

Mossman *et al*

SUPPLEMENTARY INFORMATION

Supplementary Note 1

Supplementary Fig. 1a-d shows sequential 1D cross sections during the progression of a barrier sweep with $v_\mathrm{b} = 2$ mm s^{-1}. In this sequence, instead of completing a full sweep, the barrier is stopped after a time t_s into the sweep and σ_z is analyzed. The red (blue) line indicates atoms occupying the $|\uparrow\rangle$ ($|\downarrow\rangle$) spin state. These experimental cross sections in panels (a-d) are averaged over 5 independent data runs at the same time into the sweep. In this sequence, $t_\mathrm{s} = 98$ ms corresponds to a sweep that ends at the center of the BEC.

In Supplementary Fig. 1a, the barrier just reaches the left edge of the BEC, and the atoms have a majority component in the $|\uparrow\rangle$ state. As the non-magnetic barrier starts to interact with the atoms, a portion of the atoms occupying the $|\uparrow\rangle$ state are transferred into the $|\downarrow\rangle$ state. For this barrier velocity, the BEC is totally reflected by the Gaussian potential. This spin-flip process continues as the barrier continues to move through the BEC as shown in Supplementary Fig. 1c. At the end of the sweep, the $|\downarrow\rangle$ spin state has become the dominant component (Supplementary Fig. 1d) and the real-space profile is non-Gaussian in shape.

Corresponding GPE numerical simulations are shown in Supplementary Fig. 1e-h. The 1D profiles provide density distributions for each spin component along the sweeping direction. The high frequency oscillatory behavior present in the GPE simulations is not observable in experiments due to the imaging resolution. Taking this experimental "course graining" into account, we find good agreement between experimental and simulated GPE results. Further GPE numerics have been performed for a wide range of velocities, presented in Supplementary Fig. 2 for (a) interacting and (b) non-interacting systems. In both types of systems, fast barrier sweeps ($v_\mathrm{b} > 9.3$ mm s^{-1}) result in a nearly unperturbed final state, and slow barrier sweeps result in total reflection from the barrier and a spin flip when sweeping in the positive direction. As an example, we directly compare the momentum-space distributions of interacting and non-interacting systems for a $v_\mathrm{b} = 7$ mm s^{-1} barrier sweep in the positive direction. In the case of interactions, the momentum of the spin-flipped component is shifted to $k \approx 2.2\ k_\mathrm{r}$ with a momentum spread of $\pm 0.5\ k_\mathrm{r}$. In the non-interacting case, the spin is flipped with ~100% efficiency and the condensate is kicked to $k \approx 3.7\ k_\mathrm{r}$.

When the barrier velocity is decreased to ~ 1 mm s^{-1}, no spin flip occurs even for a positive barrier velocity, though the BEC is still completely reflected. In this case, the momentum peak is shifted to $k = 0.35\ k_\mathrm{r}$, which is approximately twice the sweeping speed in units of k_r. This indicates a simple single-particle reflection process due to the suppression of the spin-flip reflection channel ($R_\downarrow$) at small velocities (see Supplementary Note 2). Experimentally, this regime is difficult to observe as low barrier velocities require long sweep times, leading to noticeable atom loss from the BEC due to Raman-induced heating.

In Fig. 3e in main text, we experimentally observe a slight decrease in the spin polarization for a left moving barrier. This is attributed to Raman-induced heating in the system as quantitatively shown in Supplementary Fig. 3a. In this figure, we have verified the heating due to the Raman beams independently by monitoring the spin polarization in the absence of a barrier sweep. This was done for times t_s used during the 2 mm s^{-1} sweep (Supplementary Fig. 3a), as well as for times associated with sweep speeds in Fig. 4 of the main manuscript (Supplementary Figure 3b). We note that heating in the system systematically causes the spin polarizations to tend toward zero.

Supplementary Note 2

In Fig. 4a of the main text, we experimentally observe a spin flip in the region of slow, right moving barrier velocities and the spin polarization has a smooth dependence on velocity below a crossover velocity around 9 mm s^{-1}. The non-interacting case provided in Fig. 5 of the main text shows that the spin polarization (transmission) exhibits a discontinuous jump from $\sigma_z = -1$ to $+1$ ($T = 0$ to 1) near this crossover velocity. The crossover velocities where σ_z changes from negative to positive values are similar in both cases, indicating that the origin of the spin-flip process can be understood by analyzing the single-particle dynamics (i.e. the non-interacting case).

There is no known analytic solution for a single particle reflecting from a stationary Gaussian potential, like the one used in this work. However, using the potential $V_{b'}$ defined in Methods, we find that a potential with a similar real-space profile (inset of Supplementary Fig. 4) can numerically reproduce the spin polarization results in a non-interacting system (Supplementary Fig. 4). Scattering from this potential is analytically tractable and is characterized by a single dimensionless parameter $\nu = w_{\mathrm{b}'}^2 U_{\mathrm{b}'}/2 \gg 1 > 1/8$. This leads to a transmission coefficient T, in the absence

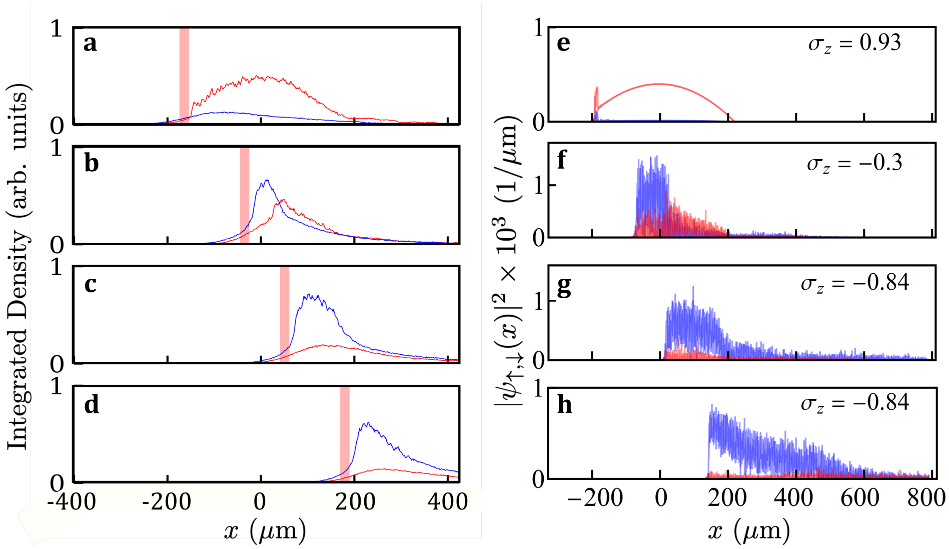

Supplementary Figure 1: **Spin flip evolution.** The optical barrier moves to the right at $v_\mathrm{b} = 2$ mm s^{-1}. Atoms originally occupying the $|\uparrow\rangle$ majority spin state are transferred to the $|\downarrow\rangle$ spin state as the sweep progresses. Red (blue) integrated densities indicate atoms occupying the $|\uparrow\rangle$ ($|\downarrow\rangle$) spin state. SO coupling parameters are the same as those in Fig. 2c of the main text. **a-d** Integrated cross sections of experimental images averaged over 5 independent data runs taken at a time, t_s, into the sweep. The thick red vertical line represents the position of the barrier prior to expansion imaging. **e-h** Corresponding integrated cross sections from numerical GPE simulations. Spin polarizations are calculated from GPE simulations found in each panel. Corresponding experimental and numerical images are taken after **a,e** $t_\mathrm{s} = 22$ ms, **b,f** $t_\mathrm{s} = 87$ ms, **c,g** $t_\mathrm{s} = 130$ ms, and **d,h** $t_\mathrm{s} = 195$ ms into the sweep.

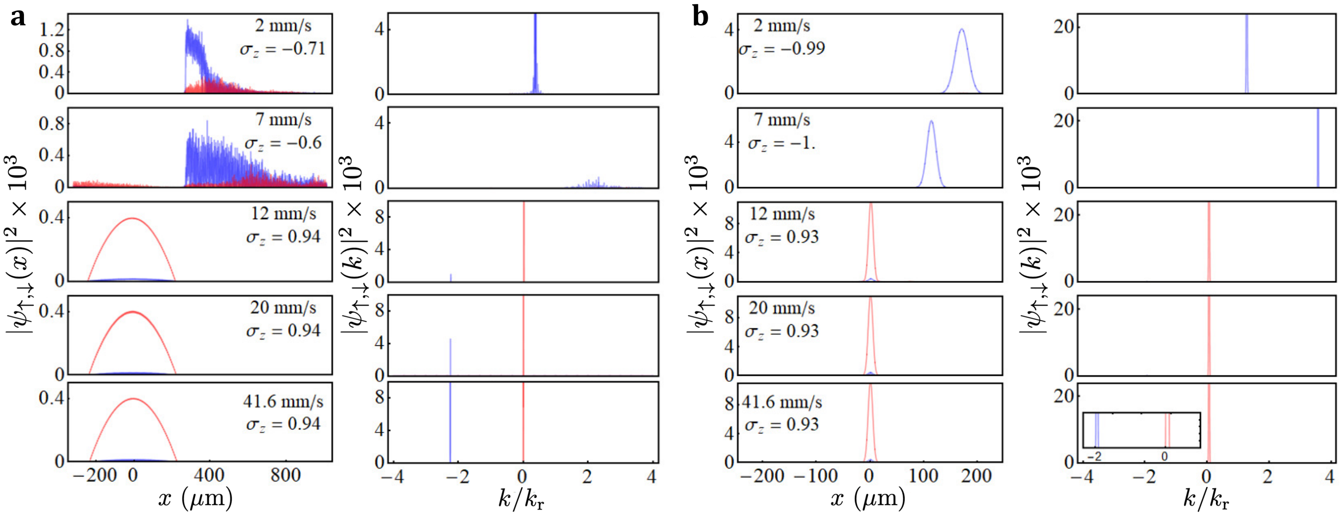

Supplementary Figure 2: **Simulations with positive velocities. a** GPE simulations for experimental parameters at increasing positive barrier velocities. Panels show the final real- and momentum-space distributions after the sweep. **b** Similar to (a) but in the single particle regime ($g = 0$). The inset of the bottom momentum-space panel shows a zoomed-in view of the momentum-space distributions.

of SO coupling, given by [1]

$$T = \frac{\sinh^2(\pi\sqrt{2\epsilon})}{\sinh^2(\pi\sqrt{2\epsilon}) + \cosh^2(\frac{\pi}{2}\sqrt{8\nu - 1})}, \tag{1}$$

where ϵ is the single-particle energy. The resulting crossover speed v_co for the transition from total reflection to total transmission is $v_\mathrm{co} = -2.85 v_\mathrm{r} \approx -16.6$ mm s^{-1}, relevant for a left sweeping barrier in our system. For a barrier moving to the right, GPE numerics show that the crossover velocity, given the experimental SO coupling parameters,

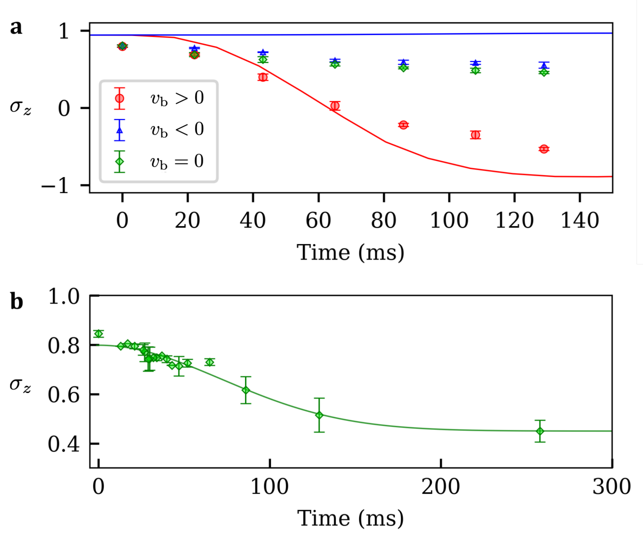

Supplementary Figure 3: **Raman-induced heating.** Measured spin polarization from Raman-induced heating. **a** Data from a 2 mm s^{-1} sweep to the right (red circles), to the left (blue triangles), and in the absence of a barrier sweep (green diamonds). **b** Data taken at times associated with barrier sweep speeds to show the reduction of spin polarization over time due to Raman-induced heating effects. Each data point taken with $v_\mathrm{b} = 0$ (green diamonds) is the mean ± s.d. of 3 experiments at each measured time in the absence of a barrier sweep.

is reduced to $v_\mathrm{co} = 1.6 v_\mathrm{r} \approx 9.3$ mm s^{-1}. These values are obtained using the non-interacting GPE model ($g = 0$).

To understand where the difference between these crossover velocities for a left verse a right moving barrier originates from, we consider the co-moving frame with respect to the barrier. In this frame, the BEC moves at $-v_\mathrm{b}$ and is scattered by a static barrier. As a first approximation, consider a single particle scattered by a given barrier in the absence of SO coupling. Depending on the dynamical energy (set by the velocity) of the particle, it will either be transmitted or reflected. For the Gaussian barrier implemented in the experiment, the dynamical energy needed for the transition between reflection and transmission is $E^0_\mathrm{co} \sim 8.4 E_\mathrm{r}$ (corresponding to a crossover velocity $v^0_\mathrm{co} \sim 2.9 v_\mathrm{r} \sim 16.8$ mm s^{-1}).

Now consider a moving barrier in the presence of SO coupling. The BEC is initially loaded into SO coupling with quasi-momentum q_i and detuning δ in the lab frame. In the co-moving frame, as discussed in the main text, the quasimomentum becomes $q_{i,\mathrm{cm}} = q_i \pm \frac{|v_\mathrm{b}|}{v_\mathrm{r}} k_\mathrm{r}$ and $\hbar\delta_\mathrm{cm} = \hbar\delta \pm \frac{4|v_\mathrm{b}|}{v_\mathrm{r}} E_\mathrm{r}$ for a left (+) or right (-) moving barrier. The band structure in the co-moving frame is depicted in Fig. 5a and b, where in (a) the atoms are moving with positive velocity and in (b) the atoms are moving with negative velocity. The dynamics here are mainly characterized by the lowest band in the presence of strong SO coupling (Ω =1.53E_r, as used in the experiment, is considered to be strong in this context). As a result, the effective dynamical energy for a left (L) or right (R) moving barrier is given by

$$E^\mathrm{L}_\mathrm{D} = \left[\left(\frac{q_i}{k_\mathrm{r}} + \frac{v_\mathrm{b}}{v_\mathrm{r}} + 1\right)^2 - 2\frac{q_i}{k_\mathrm{r}} + \frac{\hbar\delta}{2E_\mathrm{r}} - \sqrt{\left(\frac{2q_i}{k_\mathrm{r}} - \frac{\hbar\delta}{2E_\mathrm{r}}\right)^2 + \left(\frac{\hbar\Omega}{2E_\mathrm{r}}\right)^2}\right] E_\mathrm{r},$$

$$E^\mathrm{R}_\mathrm{D} = \left[\left(\frac{q_i}{k_\mathrm{r}} - \frac{v_\mathrm{b}}{v_\mathrm{r}} + 1\right)^2 - 2\frac{q_i}{k_\mathrm{r}} - \frac{\hbar\delta}{2E_\mathrm{r}} + \frac{4v_\mathrm{b}}{v_\mathrm{r}} - \sqrt{\left(\frac{2q_i}{k_\mathrm{r}} + \frac{\hbar\delta}{2E_\mathrm{r}}\right)^2 + \left(\frac{\hbar\Omega}{2E_\mathrm{r}}\right)^2}\right] E_\mathrm{r}.$$

The crossover velocity for either direction is analytically obtained by solving $E^\mathrm{L,R}_\mathrm{D} = E^0_\mathrm{co}$. Based on the parameters used in the experiment, we find $v^\mathrm{L}_\mathrm{co} \simeq 16.6$ mm s^{-1} and $v^\mathrm{R}_\mathrm{co} \simeq 9.3$ mm s^{-1}, consistent with GPE numerical simulations and experimental results quoted in the main text.

From this discussion, it is shown that the crossover velocities are primarily determined by the SO coupling parameters and the barrier shape. Although other systematic parameters like trapping frequencies and the atomic number could reduce the effective interatomic interaction strength, and thus improve the spin switch efficiency, these parameters have less of an effect on the crossover velocities. In this sense, a finely tuned system is not required to implement such a unidirectional spin switch.

In the theory developed so far, the reflection channel $R_\uparrow$ is assumed to resided on the upper band of the SO-

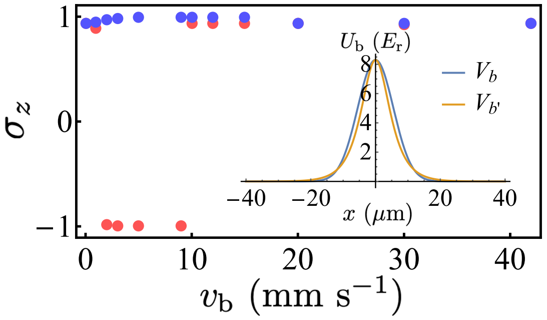

Supplementary Figure 4: **Non-interacting GPE simulations.** The dependence of the final polarization on the sweeping speed for a single particle with a modified sweeping barrier $V_{\mathrm{b}'}$ instead of a Gaussian barrier. Inset shows the potential profile for each barrier. Potential is defined in Methods.

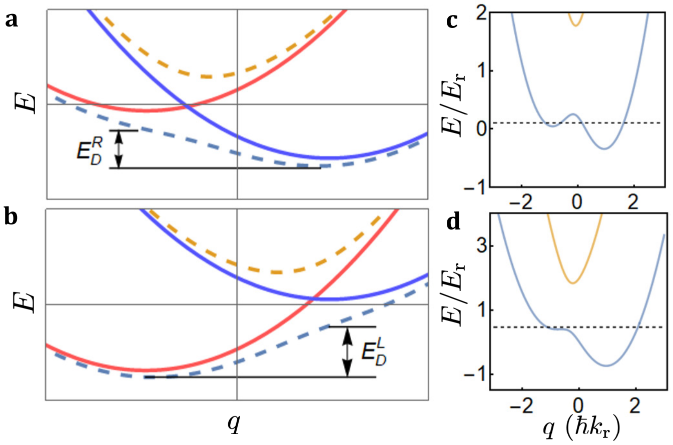

Supplementary Figure 5: **Co-moving picture. a,b** Large speed SO coupling band structures in the co-moving frame are plotted schematically for (a) $v_{\mathrm{b}} > 0$ and (b) $v_{\mathrm{b}} < 0$. Solid red and blue curves are free-particle dispersion bands for $|\uparrow\rangle$ and $|\downarrow\rangle$ and the dashed lines are SO-coupled dispersion bands with experimental coupling strength $\hbar\Omega = 1.53\ E_{\mathrm{r}}$. The Doppler shift in detuning is not plotted here. **c,d** SO coupling band structures in the co-moving frame for two positive sweeping velocities, (c) $v_{\mathrm{b}} = 0.5$ mm s^{-1} and (d) $v_{\mathrm{b}} = 1.1$ mm s^{-1}. The dashed line denotes the energy imparted by the barrier. Atoms are only able to overcome the band barrier near $q = 0$ when $v_{\mathrm{b}} \gtrsim 1.1$ mm s^{-1}.

coupled dispersion (as depicted in Fig. 5b of the main text). This is typically only true for positive barrier velocities significantly larger than a minimum critical value. For barrier velocities lower than this critical speed, atoms are unable to overcome the momentum-space barrier due to a small Doppler shift and low energy imparted from the barrier, as shown in Supplementary Fig. 5c. The resonant coupling channel, $R_\uparrow$, therefore remains open and can exist on the lower branch of the SO-coupled dispersion. As the barrier speed is increased, the momentum-space barrier flattens and more energy is imparted on the system by the barrier, causing the $R_\uparrow$ coupling channel to close, and the $R_\downarrow$ coupling channel to subsequently open, resulting in a spin-flip. For the given parameters of the experiment, the lower critical velocity is found to be $v_{\mathrm{b}} \approx 1.1$ mm s^{-1} (Supplementary Fig. 5d), consistent with GPE simulation results.

Supplementary Note 3

Based on our scattering analysis, a barrier with large potential height and a wide profile is desired for spin switching. This generates a large ν parameter (see Supplementary Note 2) and leads to a sharp transition between total reflection and total transmission, preventing an undesired mixture of reflection and transmission channels for a range of velocities. We note that even in the presence of interactions, the transition region from reflection to transmission is relatively narrow due to the large ν parameter. In addition, the barrier width w_{b} may also affect how fast atoms can be driven while still following the lower band, due to different Landau-Zener tunneling rates to a higher band for a given

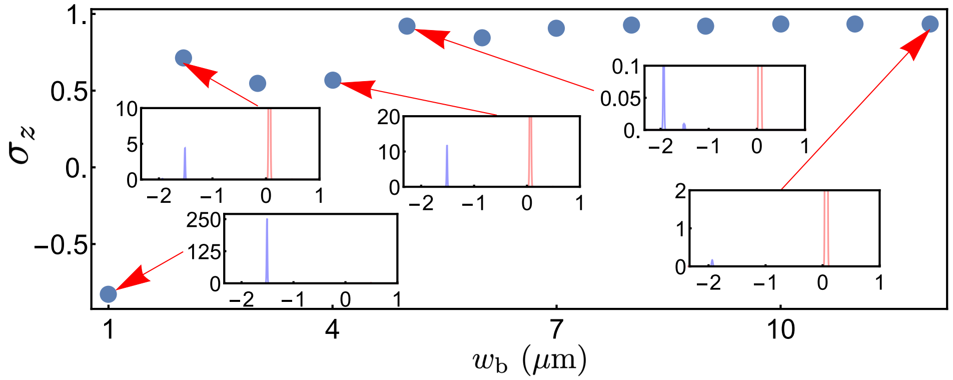

Supplementary Figure 6: **Barrier width dependence.** Spin polarization with respect to the barrier width in the single particle regime for a barrier traveling at $v_{\rm b} = +20$ mm s^{-1}. Insets show corresponding momentum-space profiles of final states. The spin flip process at this speed is accomplished solely through the $T_{\downarrow}$ channel.

sweeping speed. In general, when using a very wide barrier (or the "adiabatic" limit), we expect to observe a vanishing tunneling rate. Consequently, the spin-flip transmission channel ($T_{\downarrow}$) should be completely suppressed for sufficiently large $w_{\rm b}$.

To confirm this intuitive picture, we perform GPE simulations in the non-interacting regime over a range of barrier widths for a fixed barrier velocity, $v_{\rm b} = +20$ mm s^{-1}. Results are shown in Supplementary Fig. 6 where $T_{\downarrow}$ is completely suppressed for $w_{\rm b} > 7$ μm. In the narrow barrier limit, atoms flipped into the $|{\downarrow}\rangle$ state (blue peaks in the insets of Supplementary Fig. 6) have a negative final momentum and $T_{\downarrow}$ is observable. Recall that the barrier used in experiments has a Gaussian width in the relevant direction of 11 μm. This behavior is in agreement with predictions from the resonant condition.

In conclusion, a reasonably wide barrier leads to a sharp transition in the transmission coefficient T and, with the help of the avoided band crossing in the case of strong SO coupling, suppresses the $T_{\downarrow}$ channel. As a result, a good one-way spin switch is expected under these experimental parameters.

SUPPLEMENTARY REFERENCES

[1] ter Haar, D. *Problems in Quantum Mechanics.* (Pion Limited, London, 1975).
